# Antiferromagnetic structure in tetragonal CuMnAs thin films

**DOI:** 10.1038/srep17079

**Published:** 2015-11-25

**Authors:** P. Wadley, V. Hills, M. R. Shahedkhah, K. W. Edmonds, R. P. Campion, V. Novák, B. Ouladdiaf, D. Khalyavin, S. Langridge, V. Saidl, P. Nemec, A. W. Rushforth, B. L. Gallagher, S. S. Dhesi, F. Maccherozzi, J. Železný, T. Jungwirth

**Affiliations:** 1 School of Physics and Astronomy, University of Nottingham, NG7 2RD, United Kingdom; 2 Institute of Physics ASCR, v. v. i., Cukrovarnicka 10, 16253 Prague 6, Czech Republic; 3 Institut Laue-Langevin, 6 Rue Jules Horowitz, 38042 Grenoble, France; 4 ISIS, Rutherford Appleton Laboratory, Harwell Science and Innovation Campus, Science and Technology Facilities Council, Oxon OX11 0QX, United Kingdom; 5 Faculty of Mathematics and Physics, Charles University in Prague, 121 16 Prague, Czech Republic; 6 Diamond Light Source, Chilton, Didcot, Oxfordshire OX11 0DE, United Kingdom

## Abstract

Tetragonal CuMnAs is an antiferromagnetic material with favourable properties for applications in spintronics. Using a combination of neutron diffraction and x-ray magnetic linear dichroism, we determine the spin axis and magnetic structure in tetragonal CuMnAs, and reveal the presence of an interfacial uniaxial magnetic anisotropy. From the temperature-dependence of the neutron diffraction intensities, the Néel temperature is shown to be (480 ± 5) K. *Ab initio* calculations indicate a weak anisotropy in the (*ab*) plane for bulk crystals, with a large anisotropy energy barrier between in-plane and perpendicular-to-plane directions.

There is much current interest in developing antiferromagnetic (AF) materials, both metallic^1^,^2^ and semiconducting^3^,^4^, for applications in spintronics. As well as their widespread use for pinning magnetization in spin valve stacks^5^, large anisotropic magnetotransport effects associated with the rotation of staggered AF moments have been proposed^6^, and experimentally demonstrated^7^. The AF semiconductor LiMnAs has attracted particular interest, as it potentially offers the possibility of controlling magnetotransport anisotropies by doping or electrical gating^2^. However, the inclusion of alkali metal elements represents a challenge both for fabrication and device stability. From the known chemical trends of the I-II-V semiconductor families, it was suggested that CuMnAs may offer a stable alternative which retains similar magnetic and electronic properties^8^.

CuMnAs, which has an orthorhombic crystal structure in bulk form, can be stabilized in a tetragonal phase (Fig. 1) by molecular beam epitaxial growth on GaAs or GaP^9^. The *ab* plane lattice constant of 3.820 Å is closely matched along the half diagonal of the GaP unit cell^7^,^10^. It has been shown that tetragonal CuMnAs is antiferromagnetically ordered at room temperature, and can induce a sizeable exchange bias shift in neighbouring Fe layers^7^. The prospects for this material for applications will depend on the nature of its temperature-dependent magnetic order, which to date has been little studied. Recently, a means of rotating the AF order in tetragonal CuMnAs using electrical currents was demonstrated, which is a consequence of the particular local site symmetry of the spin sublattices^11^. Understanding of the magnetic anisotropy and its temperature-dependence is crucial for precise control of AF switching using electrical or other means.

The absence of a stray magnetic field is an appealing feature of AF materials from the point of view of spintronic memory applications, as it eliminates the possibility of interactions between neighbouring memory elements^3^. However, as a consequence the direct characterization of AF order can be challenging. Several techniques exist for probing AF order in bulk crystals, of which neutron diffraction is the principal technique for the atomic level understanding of AF order. In the case of thin films, the only established direct probe of AF order is x-ray magnetic linear dichroism (XMLD), which is the angular-dependence of the absorption of linearly polarized x-rays. This technique has been widely employed over the past two decades, for studies of exchange coupling and exchange bias in multi-layered magnetic structures^12^, and for determination of the magnitude and orientation of the magnetic moments in ultrathin AF films^13^. However, it can be challenging to distinguish magnetic contributions to the linear dichroism from the effect of charge anisotropy^14^, for example due to a lattice strain.

Here, we present detailed characterization of the temperature-dependent AF order in tetragonal epitaxial CuMnAs films. We compare the properties of a 500 nm thick CuMnAs film investigated using neutron diffraction, to those of a 10 nm film studied with XMLD. The experimental studies are combined with *ab initio* theoretical calculations of the ground-state AF structure and magnetocrystalline anisotropy energy to develop a comprehensive picture of the AF order.

## Results

### Growth and structure

**Figure 1:**
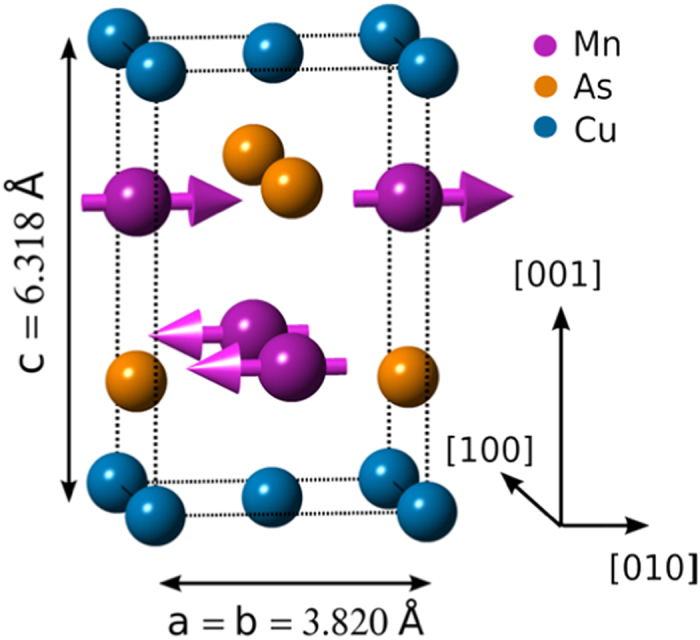
Tetragonal CuMnAs structure. Unit cell structure of tetragonal CuMnAs and spin arrangement revealed by neutron diffraction.

The CuMnAs films, of thickness 10 nm and 500 nm, were grown on GaP(001) by molecular beam epitaxy at a substrate temperature of 300 °C. The bulk magnetic properties of the 500 nm thick film were investigated using neutron diffraction. The 10 nm film, which was investigated with surface-sensitive XMLD, was capped with 2 nm Al to avoid surface oxidation. The growth rates and stoichiometry were obtained from the ion fluxes, which were calibrated using x-ray reflectivity. Superconducting quantum interference device magnetometry showed that the films contained negligible ferromagnetic secondary phases. X-ray diffraction measurements showed that both films have the tetragonal Cu_2_Sb structure (space group *P4/nmm*) shown in Fig. 1. The film and substrate follow the epitaxial relationship CuMnAs(001)[100]||GaP(001)[110], with less than 1% lattice mismatch^9^,^10^.

### Neutron diffraction

The neutron diffraction data were acquired using the D10 beamline at the Institut Laue-Langevin (ILL), Grenoble and the WISH^15^ instrument at ISIS, Rutherford Appleton Laboratory. At the ILL a single-crystal four-circle diffractometer and cryo-furnace were used. The chamber was capable of heating to 550 K in helium atmosphere. The sample was mounted such that the *ac* plane of the CuMnAs was in the plane formed by the incident beam and detector. This allowed for the detection of the CuMnAs (100) and (001) reflections without remounting the samples. At ISIS the WISH instrument was used. WISH is a long-wavelength diffractometer providing high resolution data over a *d*-spacing range from 0.7–17 Å and uses pixellated ^3^He detectors covering scattering angles from 10 to 170 degrees.

**Figure 2:**
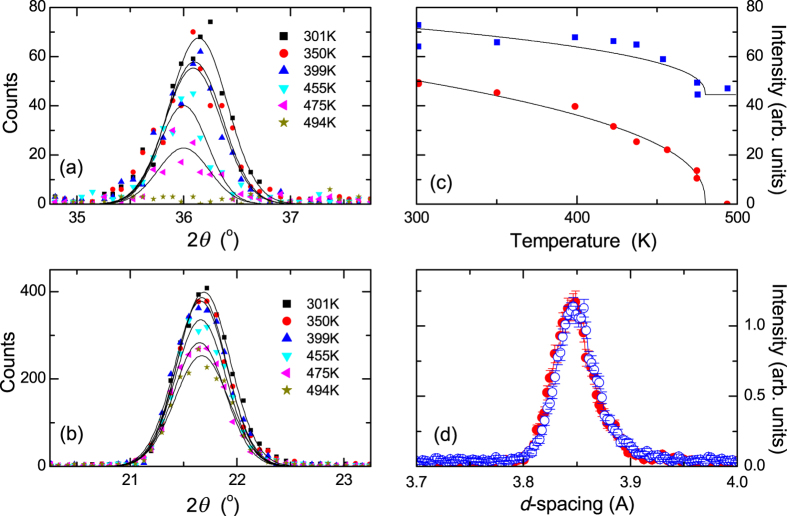
Neutron diffraction measurements of 500 nm CuMnAs on GaP(001). (**a**,**b**) Neutron diffraction peaks obtained at various temperatures for (**a**) the (100) and (**b**) the (001) atomic planes. The lines show the Gaussian fits to the data. (**c**) Temperature-dependent intensities of the neutron diffraction peaks for the (100) reflection (circles) and the (001) reflection (squares), together with power law fits. (**d**) Neutron diffraction peaks for the (100) atomic plane (filled circles) and the (010) atomic plane (open circles), normalised to the GaP substrate (220) peak.

Figure 2(a,b) show neutron diffraction peaks for the (100) and (001) planes of the 500 nm CuMnAs film recorded for a range of different temperatures. The CuMnAs (001) peak is an allowed nuclear reflection for the space group *P4/nmm* whereas the (100) reflection is structurally forbidden. This means that for the (001) reflection the intensity is comprised of a nuclear component plus a magnetic component caused by any projection of the spin axis into the (*ab*) plane (i.e. perpendicular to the scattering vector). In contrast the (100) reflection is purely magnetic and sensitive to any magnetic projection into the (*bc*) plane.

Figure 2(c) shows the respective integrated intensities of Gaussian curves fitted to the two peaks versus temperature. In the (100) reflection a clear Brillouin-like decay is seen, as expected for the temperature dependence of magnetic order. For the (001) reflection this same decay is seen above a nearly constant nuclear component resulting in a non-zero intensity at 500 K. The observed temperature-dependence is confirmation that the (100) reflection is purely magnetic in origin while the (001) reflection contains both nuclear and magnetic components.

The magnetic contribution to the structure factor is caused by the magnetic ordering perpendicular to the scattering vector. Hence, the (001) and (100) reflections are sensitive to spin projections in the (*ab*) and (*bc*) planes, respectively. The spin components within the (*ab*) plane and along the c-axis, however, are transformed by different irreducible representations of the P4/nmm space group and cannot appear simultaneously at the continuous phase transition. Thus, the experimentally observed magnetic scattering in both (100) and (001) reflections implies that the spins are confined within the (*ab*) plane. The intensity from the (100) peak is caused by the presence of antiferromagnetic domains with a spin projection along the orthogonal [010] direction. By fitting the temperature-dependence to a power law of the form (*T*_*N*_ − *T*)^2β^ , the Néel temperature *T*_*N*_ of the tetragonal CuMnAs film is determined to be (480 ± 5) K.

Figure 2(d) compares the structurally forbidden (100) and (010) reflections for the CuMnAs film grown on GaP(001), measured separately, after normalising to the substrate (220) diffraction peak. The normalisation is required because of absorption effects related to the shape of the sample. After normalizing, the (010) and (100) intensities are consistent with one another, indicating equal projections of the local moments along the [010] and [100] orientations. From analysis of the neutron diffraction data, normalized to a vanadium spectrum and scaled to the nuclear (200) peak, the Mn magnetic moment is estimated to be (3.6 ± 0.2) μ_B_ per atom at room temperature^9^.

### XMLD

**Figure 3:**
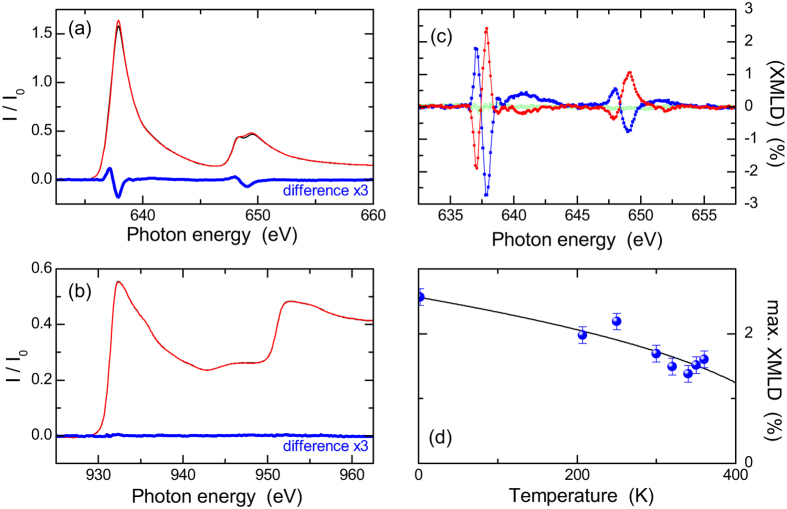
XMLD measurements on 10 nm CuMnAs on GaP(001). (**a**) Mn *L*_2,3_ x-ray absorption spectra obtained with normal incidence x-rays with horizontal (black) and vertical (red) linear polarization, and the difference spectrum (blue). (**b**) As for (**a**), but at the Cu *L*_2,3_ x-ray absorption edges. (**c**) Mn *L*_2,3_ XMLD spectra, obtained as the difference between absorption spectra for x-ray polarization parallel and perpendicular to the [100] axis (blue), the [110] axis (green) and the [010] axis (red). (**d**) Temperature-dependence of the Mn *L*_3_ XMLD (points) compared to the power law behaviour extrapolated from the neutron diffraction measurements (line).

The XMLD study was performed on beamline I06 at Diamond Light Source. The beamline’s variable polarization undulator allows the x-ray linear polarization vector to be switched between horizontal and vertical, allowing XMLD measurements without moving the sample. Mn and Cu *L*_2,3_ x-ray absorption edge spectra were obtained using the total electron yield method, with x-rays at normal incidence. In this configuration, the angle-dependence of the x-ray polarization vector is sensitive to magnetic or structural anisotropies in the plane of the layer. Figure 3(a,b) shows the x-ray absorption and linear dichroism spectra for the 10 nm CuMnAs film at temperature *T* = 250 K. The linear dichroism spectra were obtained as the difference between the absorption spectra measured with the linear polarization parallel and perpendicular to the [100] direction. A clear linear dichroism is observed at the Mn *L*_2,3_ edge, with positive/negative lobes on the lower/higher energy side of each of the spin-orbit split peaks. The absence of a linear dichroism at the Cu *L*_2,3_ edges (Fig. 3(b)) is evidence that the Mn dichroism is magnetic in origin.

In order to determine the spin axis and to rule out experimental artifacts, the dependence on relative alignments of the linear polarization and the crystal axes was investigated. Three samples, cut from the same 10 nm CuMnAs/GaP wafer, were oriented with the x-ray beam perpendicular to the sample surface, and with the x-ray polarization switched between parallel to the [100], [010] or [110] crystal axes, respectively. The Mn *L*_2,3_ XMLD spectra for the three samples at *T* = 2 K are shown in Fig. 3(c). For the first two orientations, the XMLD are similar in magnitude, but opposite in sign, while for the third orientation the XMLD is much smaller. Thus it can be concluded that for the 10 nm CuMnAs film, there is a uniaxial magnetic anisotropy such that the AF spins are preferentially oriented along one of the in-plane <100> crystal axes.

The maximum XMLD signal, defined as (*I*_*||*, *max*_ − *I*_⊥,*max*_)/(*I*_*||*, *max*_ + *I*_⊥, *max*_) where *I*_*||*, *max*_ and *I*_⊥, *max*_ are the maximum absorption signals for parallel and perpendicular alignments of the x-ray polarization and the [100] crystal axis, is shown as a function of temperature in Fig. 3(d). The XMLD signal follows the same power-law dependence as the [100] neutron diffraction peak for the thicker CuMnAs film, shown by the black line in both Figs 2(c) and 3(d). Both the XMLD and the neutron diffraction intensity are proportional to the square of the local magnetic moment. The maximum value of the XMLD signal is of comparable magnitude to that observed in ferromagnetic Mn compounds, including the Heusler alloys Co_2_MnSi and Co_2_MnAl^16^,^17^, and the diluted magnetic semiconductor (Ga,Mn)As^18^. By comparison to these compounds with known magnetic moments per atom, the XMLD signal in the thin CuMnAs films is estimated to correspond to a Mn moment of ≈2–3 μ_Β_ per atom. The precise relationship between the XMLD magnitude and the local magnetic moment depends on the measurement geometry and the degree of localization of the 3*d* valence states.

### Theory

**Figure 4:**
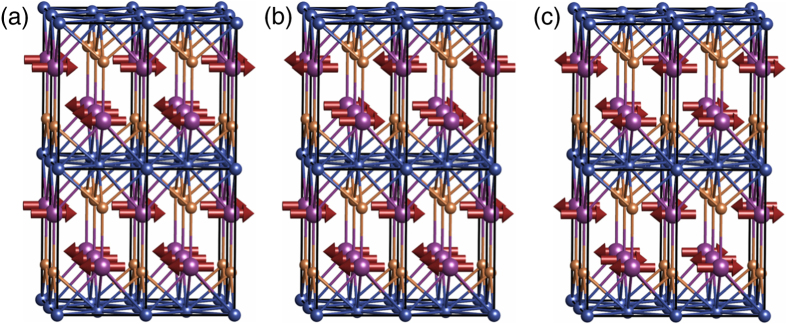
Antiferromagnetic configurations used in the calculations. Two unit cells are shown in each direction. (**a**) The experimentally observed magnetic order AF1; (**b**) AF2; (**c**) AF3.

To further clarify the magnetic structure, ground state density functional calculations were performed using the WIEN2k package^19^. We first compared total energies of structures with different magnetic orders, one ferromagnetic (FM) and three antiferromagnetic structures (AF1–3), shown in Fig. 4. For this calculation we did not include the relativistic spin-orbit coupling. We found that the AF1 structure with the lowest energy corresponds to the one observed in the neutron diffraction experiments. The AF2 structure has energy higher by 95 meV per formula unit (f.u.), the AF3 structure by 86 meV per f.u., and the FM structure by 172 meV per f.u. The large energy difference between AF and FM configurations for tetragonal CuMnAs is in agreement with the observed high Néel temperature. By way of comparison, in CuMnSb, which has a Néel temperature of 50 K, the difference between AF and FM configurations is only 25 meV per f.u.^20^.

To find the magnetocrystalline anisotropy (MAE), relativistic calculations were performed for different AF spin-axis directions with the AF1 structure. We considered [100], [110] and [001] spin-axis directions. The difference between the energies of the [100] and [110] directions was found to be less than 1 μeV per f.u., which is close to the resolution limit of the calculation. The [001] direction has energy higher by 127 μeV per f.u. To calculate the MAE, the so-called force theorem^21^ is often used in which spin-orbit coupling is not included self-consistently, but only for one iteration. For comparison we also calculated the MAE by this method. The results are very similar, except the energy difference between the in-plane directions and the [001] direction is about 30% smaller. We can, therefore, conclude that the calculations predict the easy axis is in the (*ab*) plane.

In the calculations we used the Perdew-Burke-Ernzerhof variant^22^ of the generalized gradient approximation of the exchange-correlation potential. The size of the basis was given by R_MT_K_MAX_ = 7.5, where R_MT_ is the smallest atomic sphere radius and K_MAX_ is the plane wave cut-off. We used a mesh composed of 5 000 *k*-points for the magnetic order calculations and of 40 000 *k*-points for the MAE calculations. Note that the LDA + U method could be used to improve the description of Mn *d* states. Including the U changes the results somewhat, for example the out-of-plane MAE is about 50% smaller when U is included, but the conclusions remain the same. Finally, we note that all MAE calculations were performed assuming a fixed crystal structure. Since magneto-elastic effects tend to be rather strong in AF materials, our results should be considered a lower bound for the expected anisotropy energies.

## Discussion

The neutron diffraction data on the 500 nm thick CuMnAs film are consistent with antiferromagnetic coupling of the two symmetry related Mn-sites with spins confined to the (*ab*) plane (Fig. 1). The magnetic structure of the tetragonal polymorph of CuMnAs implies a biaxial magnetocrystalline anisotropy, with spin polarization along either the <100> or <110> crystallographic directions in the (*ab*) plane. Precise determination of the spin axis direction is complicated by the presence of magnetic domains, with equal projections of the local moments along the [010] and [100] orientations. The *ab initio* calculations reveal a ground-state magnetic structure which is consistent with the experimentally observed one, and indicate a near-degeneracy of the spin axis direction in the (*ab*) plane.

For the thin CuMnAs film, a different situation is observed. The XMLD measurements indicate that the spins predominantly populate a magnetic domain oriented in one of the <100> directions in the (*ab*) plane, *i.e*. there is a uniaxial magnetic easy axis which is parallel to one of the substrate <110> crystal axis. Similar uniaxial magnetic anisotropies are commonly observed in thin *ferromagnetic* films grown on III-V semiconductor substrates^23^,^24^. While the detailed mechanism is not fully established, it is known to originate from the reduced symmetry of the bonding at the interface. For example, for Fe films on GaAs(001), the uniaxial anisotropy energy per unit volume falls in inverse proportion to the thickness of the film, characteristic of an interface effect^23^. It is likely that the uniaxial magnetic anisotropy observed for the CuMnAs film has a similar interfacial origin, and thus is suppressed in the 500 nm thick film. However, quantification of the strength of the magnetic anisotropy in an antiferromagnet is much more challenging than for ferromagnetic materials, due to the insensitivity to external magnetic fields.

The temperature-dependence of the neutron diffraction peaks indicates that the Néel temperature *T*_*N*_ of tetragonal CuMnAs is (480 ± 5) K, compared to ≈350 K for CuMnAs with orthorhombic structure^8^. Extrapolation of the temperature-dependence of the XMLD signal indicates that *T*_*N*_ of the 10 nm thin film is not significantly different to the bulk value. In addition, both the neutron intensity and the XMLD vary continuously over the observed temperature ranges.

To conclude, the neutron and x-ray measurements and *ab initio* calculations demonstrate that the spin axis in antiferromagnetic tetragonal CuMnAs films is confined within the (*ab*) plane. A uniaxial magnetic anisotropy favouring one of the <100> orientations is dominant in 10 nm thin films, giving way to a biaxial magnetic anisotropy in thicker films. The Néel temperature of (480 ± 5) K is consistent with the calculated large energy difference between FM and AF spin configurations.

## Additional Information

**How to cite this article**: Wadley, P. *et al*. Antiferromagnetic structure in tetragonal CuMnAs thin films. *Sci. Rep*. **5**, 17079; doi: 10.1038/srep17079 (2015).
